# Chicago Medical Society

**Published:** 1887-04

**Authors:** 


### CHICAGO MEDICAL SOCIETY.

*Stated Meeting, March 7th, 1887.*—THE PRESIDENT, EDMUND J. DOERING, M. D., IN THE CHAIR.

PROFESSOR FRANKLIN H. MARTIN read a paper entitled,

### HYSTERO, NEURASTHENIA, OR NERVOUS EXHAUSTION OF WOMEN.

After enumerating the symptoms of this disease the author discussed the methods of securing the requisites in treatment viz.: rest, proper feeding, seclusion and sleep. To carry out these methods successfully the physician must first secure full control and the confidence of his patient. The details of nursing, feeding, the application of massage and electricity must be in the hands of well-trained assistants. Dr. Martin cited the details of the management of a case to illustrate the details of the treatment to be followed in such cases, which in nearly all respects was similar to the S. Weir Mitchell treatment.

DR. J. G. KIERNAN said: He regarded the name adopted as a somewhat useless addition to disease-nomenclature, for it seems that most of the symptoms given are not characteristic of any particular disease other than the common form of nervous prostration occurring in women. Most of the symptoms given as regards the vagina, uterus, etc., are such as one expects to find secondary to nervous exhaustion in females. Dr. Martin has not outlined the difference between a condition of neurasthenia, which occurs in a female of previously sound constitution both from heredity and other standpoints, and the condition of the woman whose ancestors have been the victims of neurosis or hereditary disease, or who has acquired a neurotic constitution. In the first of these forms a great deal can be done by combating the exhaustion, and no better system can be given than that which has been outlined by Dr. Martin. In the last type this treatment is worse than useless. A number of patients whom S. Weir Mitchell had treated afterwards came to the private insane hospital to which Dr. Kiernan was at one time physician. With this class of patients the trouble is with the brain, and in most it is congenital. In them the *ego* is of superlative development; they are perfectly willing to be petted and fussed over, but that simply tends to develop the egotism in certain directions. In this class of cases massage is peculiarly noxious, since in many of them there is a sexual element which is likely to be increased by massage, as has been pointed out by Marrell and others. In regard to medicinal treatment, Dr. Martin has not indicated the danger of inducing habits in some of these cases by the prescription of morphine and alcohol, as Dr. Mary H. Thompson some years ago pointed out in this society. Many a neurasthenic person has become the regular inmate of an inebriate asylum or home for opium-habitues, because some physician has prescribed opium or alcohol for the treatment of insomnia.

PROFESSOR A. E. HOADLEY read a paper on

DEEP-TUBING OF THE LARYNX AS A SUBSTITUTE FOR INTUBATION, WITH A REPORT OF NINE CASES.

In these cases the tube was introduced so that the head of the tube rested within the larynx. Eight of the cases were able to drink consecutive swallows immediately after the operation. While all the cases were fatal, the deep intubation gave the greatest relief. Professor Hoadley believed the most objectionable feature in the O'Dwyer method is the projection of the head of the tube over the top of the larynx, interfering with deglutition and exciting cough. This he proposes to obviate by "deep tubing," which he accomplishes by modifying the tube as follows: shortening the tube to nearly the length of the larynx, making the head of the tube conform more nearly to the interior of the upper part of the larynx. Making the upper surface of the head of the tube cup shaped to facilitate the introduction of the extractor, having that portion of the posterior border of the tube corresponding to the arytenoid cartilages stand on a plane anterior to that of the rest of the tube, so that pressure at this point will be slight, making the obturator three-eighths of an inch longer than the tube to facilitate the deep introduction of the tube. The long tubes, owing to the mobility of their lower ends, produce irritation in the trachea, exciting cough and pain.

PROFESSOR F. E. WAXHAM opened the discussion, and said he took that occasion to present a bronchocele which produced death by pressure on the trachea. The history of this case, briefly, is this: The patient was a girl 14 years old; she had been troubled with a goitre for about a year, which increased rapidly in size, and the people observed that it was at times larger than at others, more particularly after the child had taken cold; but still the goitre gave no inconvenience until three days before death, when it is stated the child had taken a slight cold and the tumor became larger. With increase in the size of the tumor and the consequent pressure upon the gland by the sterno-thyroid muscles, great dyspnœa resulted. The patient was attended by Dr. J. G. Berry, who recognized the true condition, and on the third day called Professor Waxham to perform intubation. The child was in most desperate condition; indeed, it was moribund, the pulse was feeble, rapid and thready, hypotastic congestion had already occurred in the lungs, and the child was semi-comatose. While in this condition the largest size intubation tube was introduced into the trachea, but it failed to give relief simply because the tube was not of sufficient length. It would not reach through the stricture, and from the pressure of the gland, as rapidly as it was pressed down in position so rapidly would it be pressed up again. The child was so near dead that tracheotomy was thought to be useless. To meet such emergencies he had a much longer tube constructed, one that will pass entirely through such a stricture and consequently give perfect relief. The object in performing intubation in cases of bronchocele would be to give immediate relief, and to give time in which we might perhaps reduce the tumor by electrolysis or the internal and external use of iodine; or these measures failing, we could then leisurely enucleate the gland. Bronchocele may produce death by pressure laterally, or by suffocation from pressure upon the trachea anteriorly; or a lobe of the gland may develop between the œsophagus and trachea and produce pressure posteriorly. In this case the pressure was exerted laterally. Coming more particularly to the discussion of the paper presented to-night, he thought it should not go without a few words of criticism. As intubation has been performed nine times without success by this new procedure, he would feel that there is little to recommend it. First, in regard to the position of the tube; the author recommends that the tube be reversed, and instead of placing it with the beveled surface forward and the shoulder projecting backward, he recommends the tube turned so that the shoulder will project forward and the beveled surface backward. A case has been reported in which there was complete perforation at the base of the epiglottis from the pressure of the beveled surface of a straight tube. The most nearly perfect tubes are now made with the upper surface curved slightly backwards so that no pressure will be exerted upon the anterior wall. If we turn the tube around and place it with the projecting shoulder forward, how much greater must be the danger from ulceration. Again, he said, the author refers in his illustration to a tube with a shoulder broad posteriorly, a tube that was discarded some time since, for it has been found that a tube with an olive-shaped head fits the larynx much better than the old tube. He believes that the author is mistaken about the head of the tube riding high and resting upon the arytenoid cartilages. It depends altogether upon the judgment used in the selection of the tube. If a tube is used larger than is appropriate for the age of the patient, it will be found that while the tube can be passed into the trachea without difficulty, yet the head will not sink into the larynx, and then it will ride high and swallowing becomes very difficult, but if the tube has an olive-shaped head and is appropriate to the age, then it will sink into the cavity of the larynx, resting upon the vocal cords, and swallowing is attended with but little difficulty. In regard to the length of the tube, it would seem to me that little would be gained by making it shorter; indeed, this was one of the reasons of Buchot's failure thirty years ago. One can readily see if we use a short tube, one simply projecting through the larynx, there would be much greater danger from detachment of false membrane below the tube. He said that in offering these criticisms it was with no unkind intent, or for the purpose of discouraging attempts at improvement. He said, also, that whilst he had performed this operation over one hundred times, and with a success rarely attained by tracheotomy, yet he does not consider the instruments perfected.

PROFESSOR CHRISTIAN FENGER said he would not say anything about intubation of the larynx according to Dr. Hoadley's method, further than if there is a possibility of avoiding the difficulty in swallowing, then that is a very important point. And if a change in the position and shape of these tubes will help us to avoid that, it will make intubation still much more valuable than it has been heretofore. But he would say a few words in regard to goitre, as Dr. Waxham had shown the very interesting specimen, and as this is of great importance. As Dr. Waxham says, pressure from the goitre on the trachea causes the so-called sabre-sheath trachea with the subsequent stenosis, either from pressure or from softening of the rings from pressure and atrophy. And that is not the only danger in goitre. There are goitres where the trachea is perfectly open, and still a too-often fatal dyspnœa occurs. Those are the cases where the recurrent laryngeal nerve is affected by pressure from the tumor so as to cause symptoms of posticus paralysis. Thus we see cases where extirpation of the goitre has been made to relieve respiratory difficulties. We see that thus the respiratory difficulty remains in spite of the removal, sometimes for a time, sometimes forever; the latter when no repair of the nerve is possible. Besides these two classes of danger to goitre patients, there is a third met with, of course only in severe cases. It consists of a spasm of the glottis severe and continuous enough to terminate in sudden death. How this spasm originates is as yet unknown. The authors who have paid most attention to this subject feel inclined to believe that pressure from the goitre on the pneumo-gastric nerve causes irritation of the vagus center of the brain, and reflex contraction of the muscles of the glottis. These are the cases where goitre patients in apparently good health, with no dyspnœa, or with a slight occasional attack, will die suddenly. For instance, a patient is well and around amusing himself during the day, and in the night a sudden dyspnœa sets in and the patient dies before anything can be done. Intubation with the long tube in a goitre patient shown by Dr. Waxham may be of very great importance as a means of relieving suffocation-attacks in goitre.

PROFESSOR FERDINAND HENROTIN said his position is very strongly in favor of that taken by the author of the paper; his experience has been directly the opposite of that of Dr. Hoadley. He had only had a few cases, but if they are of any importance in elucidating the point, he would take pleasure in giving them. He believes in having the tube facing in the way mentioned in the paper. His first case was about a year ago, the patient being brought to him from out of town. As it was his first case he took particular pains to follow the regular method as pointed out by those who had introduced it, and so introduced the tube with the upper face looking forward and upward, that is, the upper plane at the entrance. The child threw up the tube within ten minutes. He immediately replaced it, and while the patient remained in the office he was perfectly free in his breathing and entirely relieved from stenosis. In spite of endeavors to have him remain, he was taken to his home in the country, the tube remaining in place. About eight o'clock the next morning he coughed up the tube. He was all right for an hour, then he began to choke up again, and in another hour died from stenosis.

In another case he had immediately after, there was no relief. He introduced the tube four different times; he had it in the larynx and it remained in positition, but the child was not relieved. He had intubed a third child, and it died.

The three cases were all with the tube in the way mentioned by Dr. O'Dwyer, and they all died. Following that he had three successful cases, and the tube was introduced the other way, facing in the other direction. In one of the cases he had no particular trouble in taking the tube out, but he had more or less trouble in introducing it, a little more than the other way. These cases all recovered. He had a case that recovered, a child of seventeen months, who retained the tube four days and coughed it up himself. Following that he again resumed the method of Dr. O'Dwyer, and placed the tube in the other direction, and that child died. In another case he was called to place a tube, and introduced it without difficulty; a little less than twenty-four hours after putting in the tube, according to the O'Dwyer method, it was coughed up by the patient. He then replaced the same tube, facing the other way, and the patient had no difficulty as yet in retaining it. He had four cases intubed by the O'Dwyer method, with four deaths; three cases with the tube turned the other way, all recoveries; probably a coincidence to a great extent. The eighth case has not progressed far enough to state whether the child would recover.

In regard to the advantages of this method, he had never had but one tube coughed up that was put in in that way, but the child recovered. The tube seems to hold much better, it sinks into the larynx and in most cases disappears, so that one cannot feel it with the finger, while if it is turned the other way one can feel the edge of it with the finger.

So far as the extraction of the tube is concerned, except in two instances, he had no particular difficulty. As regards swallowing liquids, he thinks it is speaking strongly to say that the patients can swallow. He feeds his patients on semi-solids, that slip down easily, and gives little pieces of ice to quench the thirst.

The disadvantage that attaches to the short tube is that the expulsive effort at coughing frequently causes it to be coughed up. The length and weight of the tube must be an element in keeping it in place. He believes that too small tubes are being used. He uses the second size in a child of four years.

PROFESSOR E. FLETCHER INGALS said he had no practical experience in putting in the tube the wrong way, and does not know how well it may turn out, but anatomically it is certainly wrong unless the shape of the tube be modified. It may be that with the head of the tube modified it would sink into the larynx, as recommended by the author of the paper; but with the tube as constructed by Dr. O'Dwyer it does not seem a safe procedure to insert it wrong side foremost, for fear of pressure upon the epiglottis causing ulceration. As to the effects of deglutition, if the tube fitted and would set deep in the larynx and remain there, of course the patient could swallow more readily. It seems to have been the experience of the last speaker that these patients do not always swallow readily even when the tube is seated deeply in the larynx, and Professor Ingals inclined to believe that unless some device can be invented to prevent the entrance of fluid it is better that the patient should take none. Where he had introduced the tube it has been for other physicians, so that he had usually been unable to watch the subsequent treatment; but in those that had recovered he had insisted that they should drink absolutely nothing. The child will want to drink very badly after the first twenty-four hours, but it is not a necessity for a few days. If no fluid is allowed the patient is not nearly as likely to have bronchitis or pneumonia as when it is permitted to drink. The child may have ice, it may have enemas, and may have soft solids in abundance. As to the length of the tube, he agreed with the last speaker that the long tube is the better. One of Buchot's mistakes was that he made his tube too short. But the principal reason for his failure was that he attacked tracheotomy; he not only wanted to introduce his tube, but he wanted every one else to stop tracheotomy. He inquired what success Dr. Waxham had had with the rubber collar in preventing the entrance of fluids into the air passages.

DR. JOHN W. NILES said his observation leads him to think that it does not make very much difference which way the tube is introduced as regards the patient's ability to swallow fluids. In neither way can this be done without exciting cough. In the two cases in which Dr. Henrotin had introduced the tube for him he introduced it the wrong way, and they swallowed fluids better than it had been expected they would. The next case Dr. Niles introduced it the wrong way. The child had more difficulty in swallowing than the others had. On the third day he coughed the tube up, and when reintroduced it was in the way recommended by Dr. O'Dwyer, and there was a marked improvement in his ability to take liquids. In fact, he swallowed better than the other two. He succumbed to the disease six days after the tube was first introduced.

PROFESSOR F. E. WAXHAM said, in regard to the rubber attachment which he had recently devised, he believes it to be a great improvement. Some may not desire to use the artificial epiglottis attached to the rubber collar, in which case it can be removed and the tube will still be a great improvement, inasmuch as, the head being small and surrounded by the rubber, it will fit the larynx more perfectly, there will be less irritation, and ulceration will be impossible. He believed that he recently saved two little patients in which this device was used, and had also employed it in other cases with advantage. He agreed with Professor Ingals that it is best to keep water from the little patients as far as possible. Let a small piece of ice be placed in a cloth and held in the mouth. This relieves the urgent thirst without causing the frequent coughing that arises from the endeavor to swallow water.

PROFESSOR HOADLEY, in closing the discussion, said: As mentioned in the paper, he had started out in the matter of intubation on his judgment as to the position the tube was designed to take in the larynx, for he had not given the subject sufficient attention to remember that the tube was designed to rest high in the larynx, and his experience has been so pleasant, so far as the non-interference with the function of the throat is concerned, that he decided in his own mind that deep tubing is preferable to intubation. The cases that he had tubed in that manner were all bad ones; not in a single one could a favorable prognosis be given. They were all promptly relieved, and that was all one could expect to attain from the tubing. The only advantage that the long tube can have over the short one is its weight, except in rare cases, and then its length is of advantage. In the majority of cases of laryngeal stenosis the obstruction is entirely in the larynx, and when it does extend down into the trachea the probabilities are that the child will die of continued extension, and in such a case the long tube can be introduced. In the nine cases tubed in this manner he had used the long tube, so that he could not say whether the short one would have relieved the stenosis. In one autopsy where there was laryngeal diphtheria there was no diphtheritic exudation in the trachea, so that the extension of the tube to the seventh ring was unnecessary. In reference to the position of the tube resting against the epiglottis, as in the O'Dwyer method, he would state that the epiglottis folds down and bears first upon the shoulder of the tube, then closes its orifice, but it cannot perfectly close the larynx. Dr. O'Dwyer has had ulceration of the epiglottis with perforation. One might think it would be a great deal worse to turn the tube around—that the pressure would be greater upon the epiglottis; but when the tube is turned around it goes half an inch lower, and the projection of the tube that is supposed to touch the epiglottis does not touch anything, and the mucous membrane is not pressed upon at all. Introducing the tube in the O'Dwyer method it will not go into the larynx; one can push it down and still it can be felt above the larynx, so when the epiglottis folds over, it must necessarily come against the tube. By turning the tube around, sinking it well into the larynx, it goes down so far that in a small larynx one cannot touch it with the finger; it goes down as far as the attachment of the apex of the epiglottis with the thyroid cartilage. The epiglottis folds down over the larynx, the laryngeal tube is still free below it, resting on the vocal cord, so that it can produce no ulceration of the epiglottis. As to the vocal cords, Dr. O'Dwyer says that ulceration is rare in that situation. That is because the pressure is slight, and not attended by friction, as in the epiglottis. Northrup says one must press the tube well down into the larynx. In doing this it rests against the epiglottic cartilage and its projection presses back against the arytenoid cartilages, pressing them wide apart in the most disagreeable manner. It is like putting the right foot into the wrong boot. It does not fit or rest easy at any point. Turn the tube around and it rests comfortably in the larynx without one point of pressure; the only pressure induced is that of swallowing, which is not sufficient to produce ulceration.. To facilitate extraction he had constructed a cup-shaped depression on the head, instead of the oval convex surface with the hole directly in the summit. With the oval head, unless one strikes the aperture itself, one is not likely to find it, as the extractor will invariably glide to the outside of the tube. With the cup-shaped head, partly from the tube being surrounded by the larynx, the extraction is greatly facilitated. His patients, with the one exception mentioned, had all been able to drink fluids; two or three swallows without coughing. Occasionally there was some irritation; they would drink a large swallow and cough, immediately drink again and then cough, but not one refused to drink. The object of the rubber collar with its valve-like attachment is to close the orifice of the tube. Inasmuch as the epiglottis will close the tube by its automatic action of folding down over it, he regards the rubber top as being useless; not only useless, but he believes that the valve, standing up in constant apposition with the epiglottis, is a source of additional and continued irritation.

